# Comparative untargeted and targeted metabonomics reveal discriminations in metabolite profiles between *Mycoplasma capricolum* subsp. *capripneumoniae* and *Mycoplasma capricolum* subsp. *capricolum*

**DOI:** 10.3389/fmicb.2023.1294055

**Published:** 2023-12-08

**Authors:** Huafang Hao, Xiaoliang Zhang, Shengli Chen, Shimei Lan, Zhangcheng Li, Shuang Liu, Xinmin Yan, Pengcheng Gao, Yuefeng Chu

**Affiliations:** ^1^State Key Laboratory for Animal Disease Control and Prevention, College of Veterinary Medicine, Lanzhou Veterinary Research Institute, Chinese Academy of Agricultural Sciences, Lanzhou University, Lanzhou, China; ^2^Gansu Province Research Center for Basic Disciplines of Pathogen Biology, Lanzhou, China; ^3^Key Laboratory of Veterinary Etiological Biology, Key Laboratory of Ruminant Disease Prevention and Control (West), Ministry of Agricultural and Rural Affairs, Lanzhou, China

**Keywords:** metabolomics, mycoplasma, energy metabolomics, metabolite profiles, lactate accumulation

## Abstract

**Background:**

Mycoplasmas are among the smallest prokaryotic microbes that can grow and proliferate on non-living media. They have reduced genomes, which may be associated with a concomitant reduction in their metabolic capacity. *Mycoplasma capricolum* subsp. *capripneumoniae* (Mccp) and *Mycoplasma capricolum* subsp. *capricolum* (Mcc), both belong to the *Mycoplasma mycoides* cluster, are significant important pathogenic *Mycoplasma* species in veterinary research field. They share high degree of genome homology but Mcc grows markedly faster and has higher growth titer than Mccp.

**Methods:**

This study investigated the metabolites of these two pathogenic bacteria from the middle and late stages of the logarithmic growth phase through liquid chromatography–mass spectrometry–based metabolomics and targeted energy metabolomics. The multivariate analysis was conducted to identify significant differences between the two important *Mycoplasma* species.

**Results:**

A total of 173 metabolites were identified. Of them, 33 and 34 metabolites involved in purine and pyrimidine, pyruvate metabolism, and amino acid synthesis were found to significantly differ in the middle and late stages, respectively. The abundance of fructose 1,6-bisphosphate, ADP, and pyruvate was higher in Mcc than in Mccp during the whole logarithmic period. Lactate was upregulated in slow-growing Mccp. The pH buffering agent N-[2-hydroxyethyl]piperazine-N′-[2-ethanesulfonic acid] added to media effectively prevented pH reduction and increase bacterial viability and protein biomass. The multivariate analysis revealed that the two *Mycoplasma* species significantly differed in glucose metabolism, growth factor transport and metabolism, cholesterol utilization, and environmental regulation.

**Conclusion:**

The study data are beneficial for understanding the metabolomic characteristics of these two crucial *Mycoplasma* species and shedding more light on mycoplasma metabolism, and serve as a resource for the pathogenesis and development of related vaccines.

## 1 Introduction

Mycoplasmas are able to cause persistent infections and chronic disease in animals and humans. They have reduced genomes and are a well-characterized model bacterium commonly used for several biotechnology and synthetic biology studies (Gibson et al., 2010; Breuer et al., 2019; Garzon et al., 2022; Maritan et al., 2022). Mycoplasmas from different hosts (cattle and poultry) markedly differ in their metabolite steady-state levels and carbon source utilization (Masukagami et al., 2017). Bacterial metabolism is related to their pathogenesis and virulence (Richardson, 2019; Sauer et al., 2019). *Mycoplasma capricolum* subsp. *capripneumoniae* (Mccp) and *M. capricolum* subsp. *capricolum* (Mcc) are crucial pathogenic *Mycoplasma* species that cause small ruminant diseases. Mccp is the pathogenic bacteria responsible for contagious caprine pleuropneumonia (CCPP), which is widely distributed in Asia and Africa (Manso-Silván and Thiaucourt, 2019; Zhu et al., 2021). CCPP is a highly contagious and harmful respiratory disease that causes acute and severe pleuropneumonia or life-long chronic infection of goats. It thus severely damages the goat breeding industry (Iqbal et al., 2019). Mccp may also infect sheep (Teshome et al., 2018; Ahaduzzaman, 2021). Mccp was recently found to infect wild ungulates, such as Tibetan antelope and Arabian oryx (Yu et al., 2013; Chaber et al., 2014). Mcc, one of the causative agents of contagious agalactia (CA), can cause CCPP-like pneumonia symptoms similar in goats (Ostrowski et al., 2011). According to the results of Blast comparison online, Mccp and Mcc have a high genome homology of approximately 97.57% and both are closely related members belonging to the “*Mycoplasma mycoides* cluster” (Grosjean et al., 2014). Mcc grows markedly faster and has higher growth titer than Mccp (Assunção et al., 2006). However, their metabolomic characteristics remain poorly understood.

Metabolomics analysis has emerged as a crucial technique for studying microbes. It is being widely used for revealing information about the metabolome and metabolic pathways within microorganisms and reflecting their changes (Joshua, 2019; Ye et al., 2021; Joffre et al., 2022; Zhang et al., 2023). In this study, a non-targeted metabolomic approach and targeted energy metabolomic were used for the discovery and quantification of Mccp and Mcc metabolites in the middle and late stages of the logarithmic phase. The correlation among the differential metabolites and the metabolic pathways involved was analyzed and verified through *in vitro* culture. Understanding the metabolic characteristics of these two economically important species of *Mycoplasma*, as well as comprehending mycoplasma pathogenesis from the perspective of metabolism and the vaccine development are beneficial.

## 2 Materials and methods

### 2.1 Strains and culture conditions

The Mccp strain M1601 (GenBank accession no. NZ_CP017125.1) was isolated from the goat with CCPP in Gansu Province, China and characterized as an inactivated vaccine strain in China (Chen et al., 2017). The Mcc strain Ckid (California kid, GenBank accession no. NC_007633.1) served as the reference strain and was stored in LVRI, CAAS. Both strains were grown at 37°C in an aerobic environment in modified Thiaucourt’s medium (MTB) for 24–72 h as previously described (Chen et al., 2017). The protein content of cultured mycoplasma cells was measured using the Pierce BCA Protein Assay Kit (Thermo).

### 2.2 Sample collection

The frozen Mcc and Mccp cells were diluted to 10^3^ color change units per milliliter (CCU/mL). Then, 1 mL of the cell suspension was inoculated into 600 mL fresh MTB medium in a 1-L flask and incubated at 37°C. The samples were collected every 1 h (Mcc) or 3 h (Mccp), and the mycoplasma sample titers were measured using a 96-well plate, as described previously (Masukagami et al., 2017). Briefly, 180 μL MTB medium was dispensed into each well. Later, 20 μL culture was added to the first column, which was then serially diluted 10 times until column 10 (column 11 was skipped), and column 12 was used for the medium negative control. Test plates were incubated for 2 weeks at 37°C in a humid environment. The wells that showed growth at the three highest dilutions were counted, and the CCU per milliliter was calculated. Three independent experiments were conducted. The mycoplasma cells were collected in the middle and late stages of the logarithmic growth phase for metabolomics analysis. The cultures were sampled at 12 and 15 h for Mcc and 38 and 51 h for Mccp, respectively. Six biological replicates were performed for each sample.

### 2.3 Extraction of metabolomics samples

Mcc and Mccp cultures in the middle and late stages were centrifuged at 4°C, 12,000 × *g* for 30 min and washed three times in ice-cold phosphate-buffered saline (PBS, pH 7.4). The cell pellets were rapidly frozen in liquid nitrogen and stored at −80°C. Six biological replicate samples were harvested for the metabolomics analysis. The metabolites were extracted as previously described (Liu et al., 2021). In brief, the samples were lysed with pre-cooled extraction buffer (methanol:acetonitrile:water, 2:2:1, v/v/v). After the mixture was vortexed, the cells were sonicated at 53 kHZ, 350 W for 60 min in an ice-bath and placed at −20°C for 1 h for protein precipitation. The supernatant was separated through centrifugation at 14,000 × *g* for 20 min at 4°C, dried through vacuum and reconstituted with 100 μL acetonitrile water solution (1:1, v/v) and then centrifuged at 14,000 × *g* for 15 min at 4°C. The supernatant was transferred to a new tube for LC–MS/MS analysis.

### 2.4 Liquid chromatographic analysis

The collected supernatant samples were placed in a 4°C automatic sampler for liquid chromatographic separation by using the Agilent 1290 infinity LC (Agilent) ultra-high performance liquid chromatography system (UHPLC) with an ACQUITY UPLC BEH Amide (1.7 μm, 2.1 mm × 100 mm column, Waters). The injection volume, flow rate, and column temperature were 5 μL, 0.3 mL/min, and 25°C, respectively. The mobile phase A was 25 mM ammonium acetate and 25 mM ammonia hydroxide in water, and the mobile phase B was acetonitrile. The chromatographic gradient elution was performed as follows: from the start to 0.5 min, B was held at 95%, linearly decreased to 65% during the next 6.5 min, linearly decreased to 40% in 2 min, and kept constant for 1 min. Subsequently, the mobile phase B was returned to 95% in 1.1 min and held for an additional 4.9 min. Quality control (QC) samples were inserted into the sample queue to monitor and evaluate the system’s stability and the reliability of experimental data.

### 2.5 Mass spectrometry

Each sample was subjected to electrospray ionization (ESI) positive/negative modes on the Triple-TOF 5600 mass spectrometer (AB SCIEX) after separation through UHPLC as described previously (Han et al., 2020). The ESI source parameters were as follows: ion source gas 1 (Gas1): 60, ion source gas 2 (Gas2): 60, curtain gas (CUR): 30, source temperature: 600°C, and ion spray voltage floating (ISVF): ±5,500 V. TOF MS scan m/z range was 60–1,200 Da, TOF MS scan accumulation time was 0.15 s/spectra, product ion scan m/z range was 25–1,200 Da, and accumulation time was 0.03 s/spectra. For MS/MS analysis, information-dependent acquisition with a high sensitivity mode was established as follows: exclude isotopes within 4 Da and candidate ions to monitor per cycle 6. The collision energy was set at 30 eV, and the declustering potential was ±60 V.

### 2.6 Targeted metabolomics analysis

Targeted energy metabolism was detected as described previously (Xue et al., 2022). The Shimadzu Nexera X2 LC-30 AD was used for chromatographic separation. First, 15 μL of the samples was injected sequentially using an LC autosampler. The ACQUITY UPLC BEH Amide column (1.7 μm, 2.1 mm × 100 mm, Waters) was heated to 40°C under a flow rate of 300 μL/min. The mobile phase consisted of A (20 mM ammonium acetate, 5% acetonitrile, pH 9.45) and B (100% acetonitrile). The gradient started at B was held at 95% from 0 to 1 min, B was decreased linearly to 55% from 1 to 12 min; B was decreased linearly from 55 to 40% from 12 to 13 min, and B was maintained at 40% from 13 to 15 min, B was returned to 95% from 15 to 15.1 min, and B was maintained at 95% from 15.1 to 18 min. To determine the system’s stability and repeatability, a QC sample was placed before the sample queue began, after every 12 samples, and after the sample queue. The mass spectrometric analysis was performed using the QTRAP 5500 mass spectrometer (AB SCIEX) in positive/negative ion modes. The MS conditions were established as follows: source temperature 550°C, Gas1: 40, Gas2: 50, CUR: 35, IISVF: 5,500 V in a positive ion mode and − 4,500 V in a negative ion mode. The mass spectrometer was operated with a dwell time of 200 ms. Each metabolite standard (50 mg/mL) was first analyzed through LC–MS/MS to obtain the optimal multiple reaction monitoring (MRM) transition parameters and construct the metabolite MRM library. Then, the retention time of 40 energy metabolites was determined by measuring the corresponding MRM (Q1/Q3) transition individually.

### 2.7 Data processing and statistical analysis

The raw data were aligned and extracted using the XCMS software package (v3.12.0)^1^ based on the peak area and retention time. Before conducting the chemometrics analysis, the metabolite ion peak with missing values of >50% were removed, and the total peak areas of positive and negative ion data in each sample were normalized. Both ESI-positive and -negative ions with a statistical significance were merged and analyzed using the SIMCA-P 14.1 software package (Sartorius Stedim Data Analytics AB, Umeå, Sweden) with pareto-scaling. Multidimensional statistical analysis of component analysis (PCA), partial least squares discriminant analysis (PLS-DA), and orthogonal partial least squares discriminant analysis (OPLS-DA) were then performed (Han et al., 2020; Xue et al., 2022). The metabolites were conducted by MetaboAnalyst 5.0^2^ (Pang et al., 2021) and Kyoto Encyclopedia of Genes and Genomes (KEGG) web service^3^ for involved metabolic pathways and enrichment analysis. MRM data files were processed for peak finding, alignment, extraction, and filtering by using MultiQuant software (v3.0.3). Statistical significance was determined using Student’s *t*-test, and variation multiple analyses (*p* < 0.05) was performed using SPSS 17.0 version (SPSS, Inc., Chicago, IL).

## 3 Results

### 3.1 Growth differences between Mccp and Mcc

**Figure 1:**
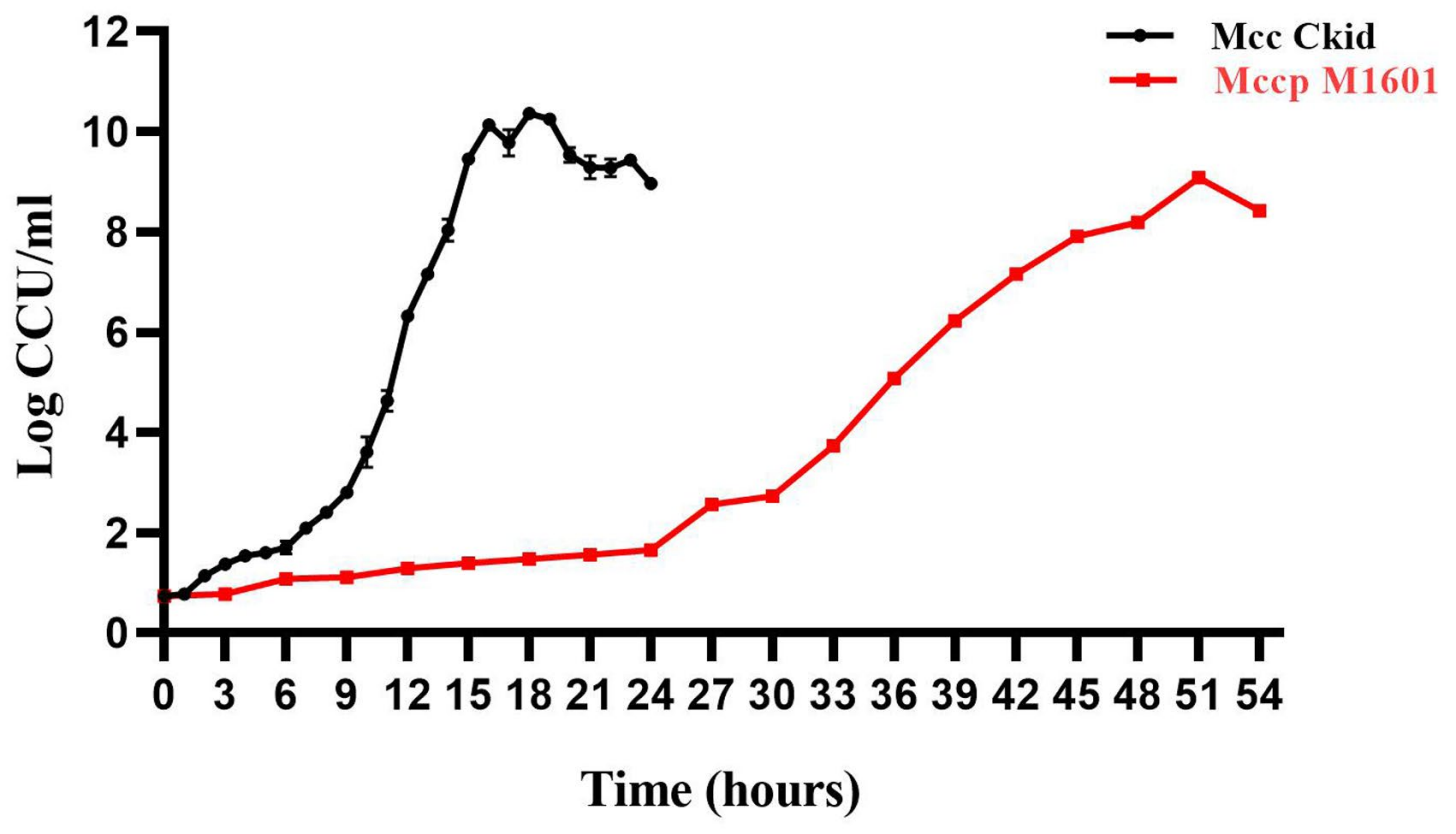
Growth curve of Mccp and Mcc in logarithmic phase. Total bacterial counts in CCU (CCU/mL) of Ckid and M1601 strains in MTB media.

The growth curves of Mccp M1601 and Mcc Ckid were compared on the basis of CCU of different times. The highest growth titer for Mcc was 2.34 × 10^10^ CCU/mL, which was 19-fold higher than that of Mccp (1.21 × 10^9^ CCU/mL). The Mcc titers during 15–30 h were all higher than the maximum titer of Mccp. Meanwhile, the Mcc strain grew quite faster than the Mccp strain. The logarithmic growth period of Mcc was lasted from 9 to 15 h, while that of Mccp lasted from 30 to 51 h (Figure 1).

### 3.2 QC and multivariate statistical analysis

**Figure 2:**
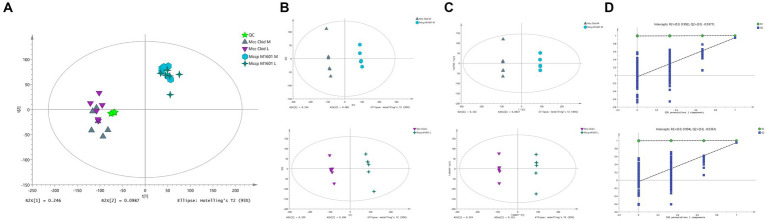
Quality control and multivariate analyses of samples. The PCA score chart of the samples. t[1] represents principal component 1, t[2] represents principal component 2 **(A)**. Supervised partial least squares discriminant analysis (PLS-DA) **(B)**. Orthogonal partial least squares discriminant analysis (OPLS-DA) **(C)**. A 200-time permutation test for evaluating the OPLS-DA model **(D)**.

QC and multivariate statistical analyses, including PCA, PLS-DA, and OPLS-DA, were performed to assess whether the data were reliable. Supplementary Figure S1 presents the total ion current (TIC) of the QC samples in both positive and negative ion detection modes. The TIC values showed a highly overlapping response intensity and peak retention time. The correlation score of the QC samples (0.963–0.999) indicated a good correlation with high accuracy data. The analysis was highly stable and repeatable. Approximately 15,043 peaks of ESI positive and 13,148 peaks of ESI negative were detected using MSDIAL software (v3.66). The obtained peaks were analyzed through PCA with Pareto-scaling following sevenfold analysis cross-validation. The samples were clustered together closely (Figure 2A), which indicates that the test had a good repeatability. The PCA score plot (Figure 2A), PLS-DA (Figure 2B), and OPLS-DA (Figure 2C) revealed a clear separation without overlap between the Mccp and Mcc groups, indicating different metabolomic profiles for them. A cluster of 200-time permutation test was conducted to validate the OPLS-DA model (Figure 2D).

### 3.3 Significant differences in metabolites between Mccp and Mcc in the middle and late of logarithmic growth phase

**Figure 3:**
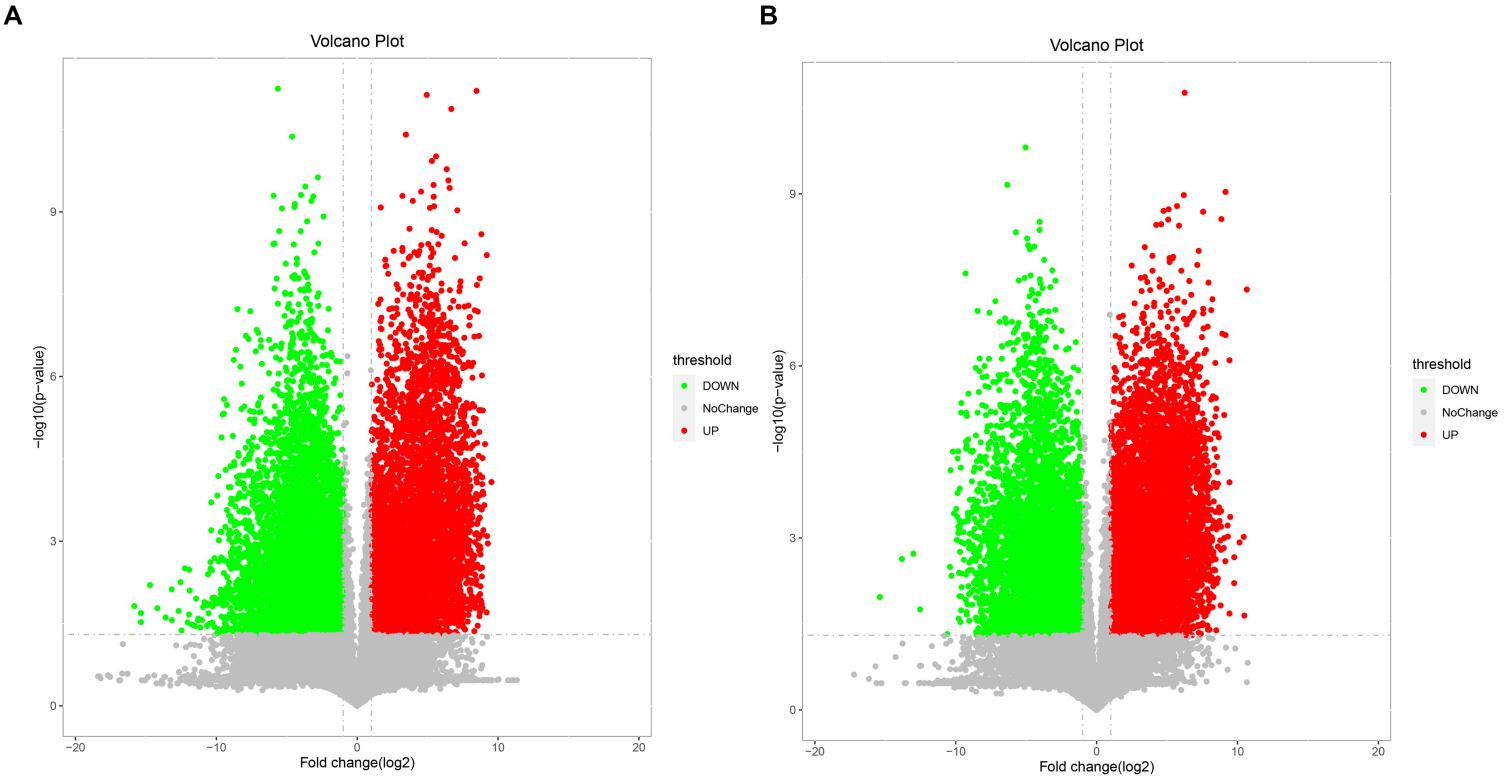
Volcano map of differential metabolites of Mcc and Mccp. **(A)** Comparison of two strains in the middle stage of logarithmic growth. **(B)** Comparison of two strains at the end of logarithmic growth. The red dots in the figure indicates upregulated metabolites, and the green dots indicate downregulated metabolites.

The differential metabolites between the Mccp and Mcc groups were analyzed through fold change analysis and *t*-test [fold change (FC) > 2 or FC < 0.5]. *p* < 0.05 was used as the screening standard in this test (Figure 3), and the volcano plot was created. Each point in the volcano map represents a metabolite.

**Figure 4:**
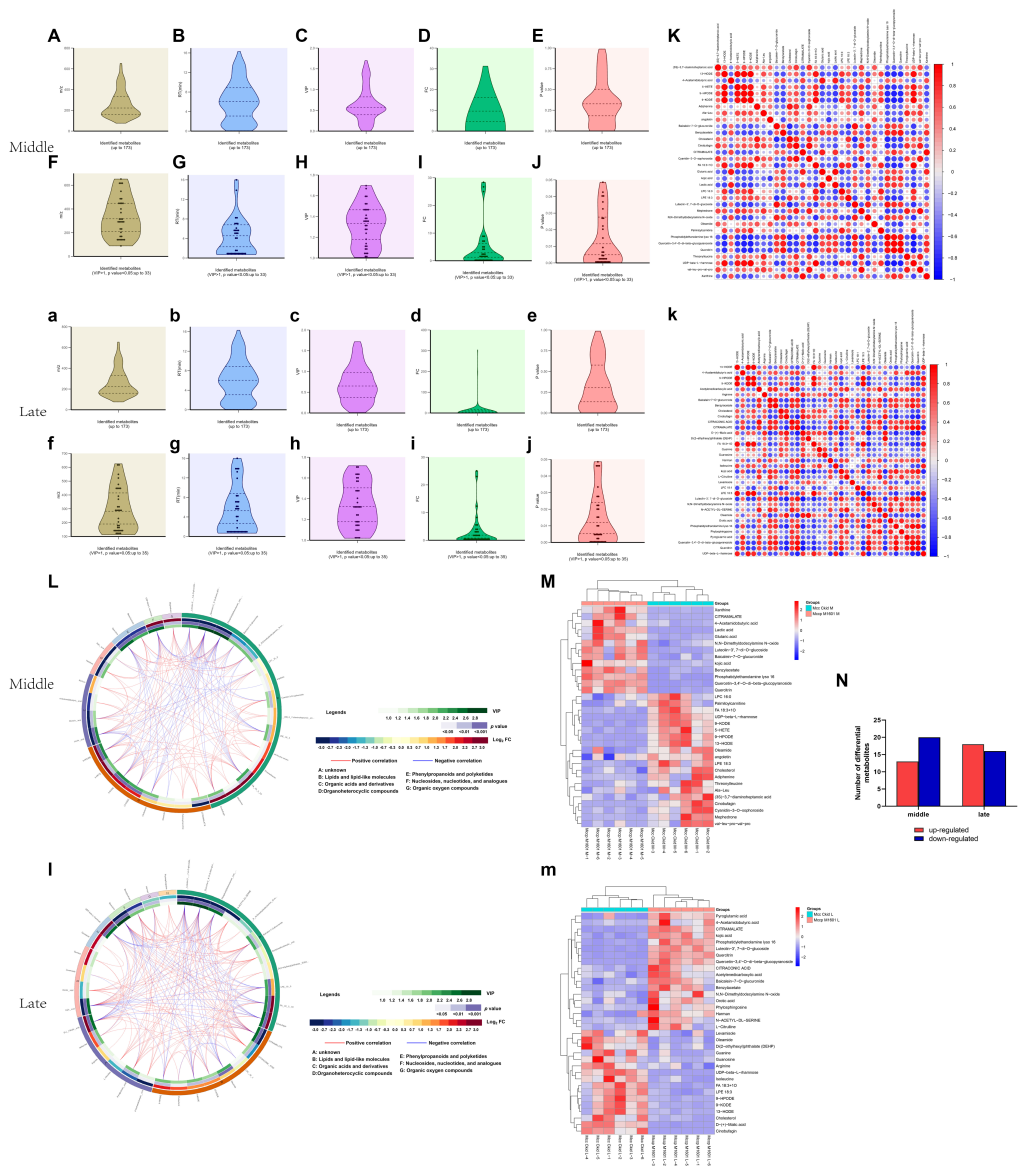
Metabolomic profiling and differential metabolite analysis. Data distributions of the mass-to-charge ratio (m/z) **(Aa)**, retention time (RT) **(Bb)**, variable importance for the projection (VIP) **(Cc)**, fold change (FC) **(Dd)**, and *p* value **(Ee)** for all identified metabolites both in the middle and late logarithmic growth phases. Data distributions of m/z **(Ff)**, RT **(Gg)**, VIP **(Hh)**, FC **(Ii)**, and p value **(Jj)** for significantly differential metabolites. The correlation analysis among significantly differential metabolites **(Kk)**. −1 < Pearson’s correlation coefficient (R) < 1. Chord diagram based on the calculation of Pearson’s correlation coefficients for significantly differential metabolites **(Ll)**. The hierarchical clustering results of significantly differential metabolites between Mcc and Mccp **(Mm)**. Numbers of metabolites upregulated (red) and downregulated (blue) in Mcc and Mccp **(N)**. The majuscule and lower case indicate middle and late logarithmic growth phase results, respectively.

In total, 173 metabolites were identified in the Mccp and Mcc logarithmic growth phases (Supplementary Table S1). Figures 4A–Ea–e exhibit data distribution of m/z, retention time, variable importance in the projection (VIP), FC and p for all metabolites in middle and late logarithmic phases. The metabolites satisfying VIP > 1 and *p* < 0.05 criteria were selected as significantly different metabolites (Figures 4F–Jf–j). Compared with Mcc, 13 upregulated and 20 downregulated differential metabolites were observed in the middle logarithmic phase of Mccp, including 10 fatty acids and derivatives, 3 lipids, 6 sugars, 5 amino acids and derivatives, and some other metabolites. Meanwhile, 18 upregulated and 16 downregulated metabolites were found in the late logarithmic phase of Mccp, including 13 fatty acids and derivatives, 4 lipids, 5 sugars, 6 amino acids, and some other metabolites (Supplementary Table S2; Figure 4N).

### 3.4 Correlation and clustering analysis of differential metabolites

The correlation analysis was conducted based on the calculated Pearson’s correlation coefficients and HMDB.^4^ Figure 4Kk presents the positive (red) and negative (blue) correlations between differential metabolites. Additionally, the HMDB analysis divided differential metabolites into seven categories (Figure 4Ll). Hierarchical cluster analysis of the samples of each group was performed on the basis of the concentration of the screened significantly different metabolites. The results revealed that the different metabolites of each group were clustered together (Figure 4Mm). Compared with Mcc, lactate and xanthine concentration was significantly upregulated, whereas cholesterol was significantly downregulated in Mccp during the middle logarithmic phase. However, in the late logarithmic phase, citrulline was significantly upregulated, whereas guanine, arginine, isoleucine, cholesterol, and malate were significantly downregulated, which indicated that positive correlations existed among the downregulated substances. Lactate and xanthine exhibited the most positive correlation with glutaric acid and citramalate, respectively, but they both exhibited a negative correlation with UDP-beta-L-rhamnose.

**Figure 5:**
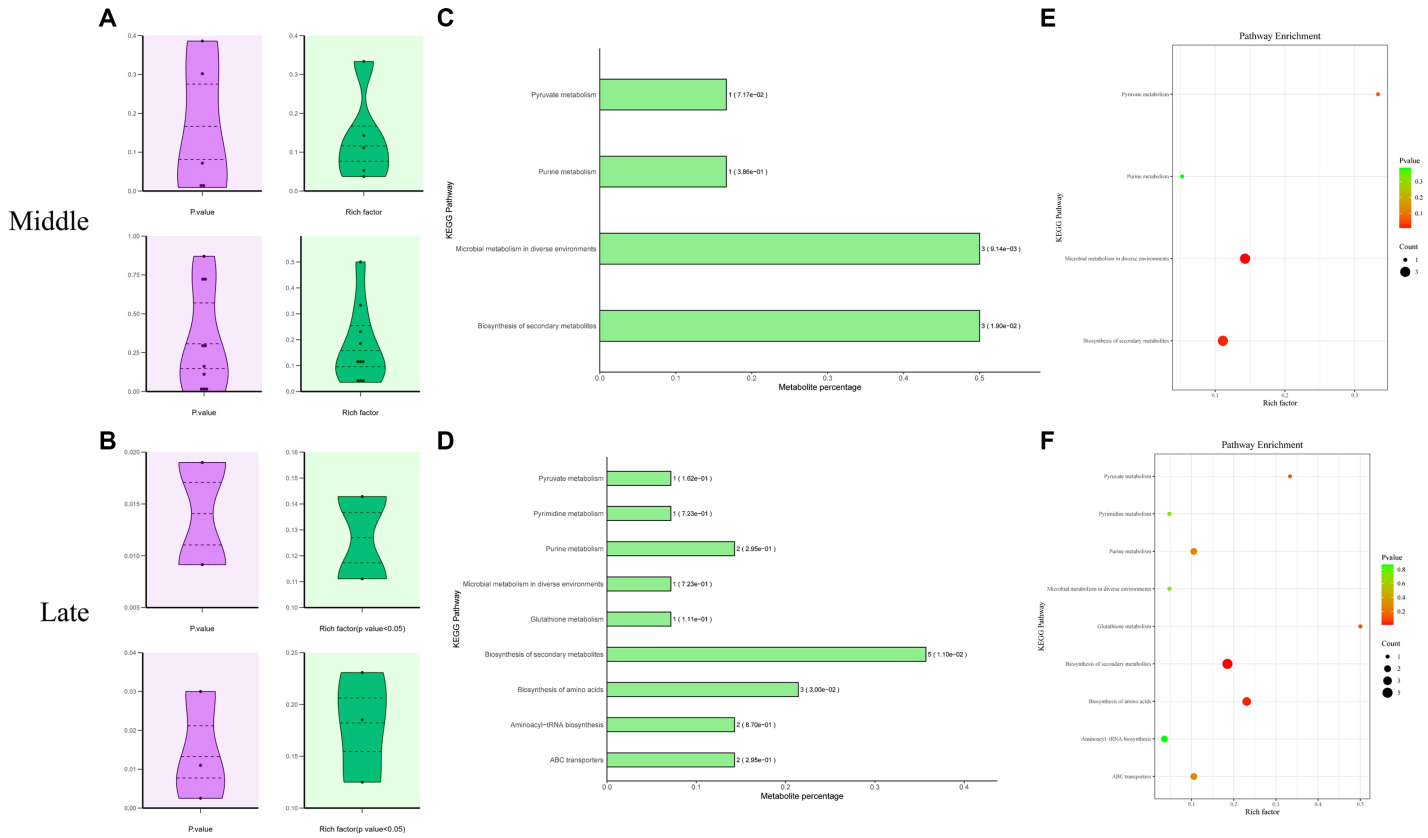
Enrichment analysis of KEGG metabolic pathways related to significantly differential metabolites in Mccp and Mcc. Distribution of *p* values and rich factors corresponding to all identified or significantly differential KEGG pathways **(A,B)**. A histogram of significant KEGG pathways **(C,D)**. Each bar is followed by the number of significantly differential metabolites with the corresponding p value. Metabolite percentage = Count/List Total. A bubble plot of significant KEGG pathways **(E,F)**.

The KEGG Metabolome Database and MetaboAnalyst 5.0 were used to identify the corresponding pathway database related to the significantly differential metabolites. These metabolites are involved in microbial metabolism in diverse environments, pyruvate metabolism, purine metabolism, pyrimidine metabolism, glutathione metabolism, biosynthesis of amino acids, secondary metabolite biosynthesis, aminoacyl-tRNA biosynthesis, and ABC transporters (Figure 5).

### 3.5 Energy metabolites were quantitatively analyzed between Mccp and Mcc

**Figure 6:**
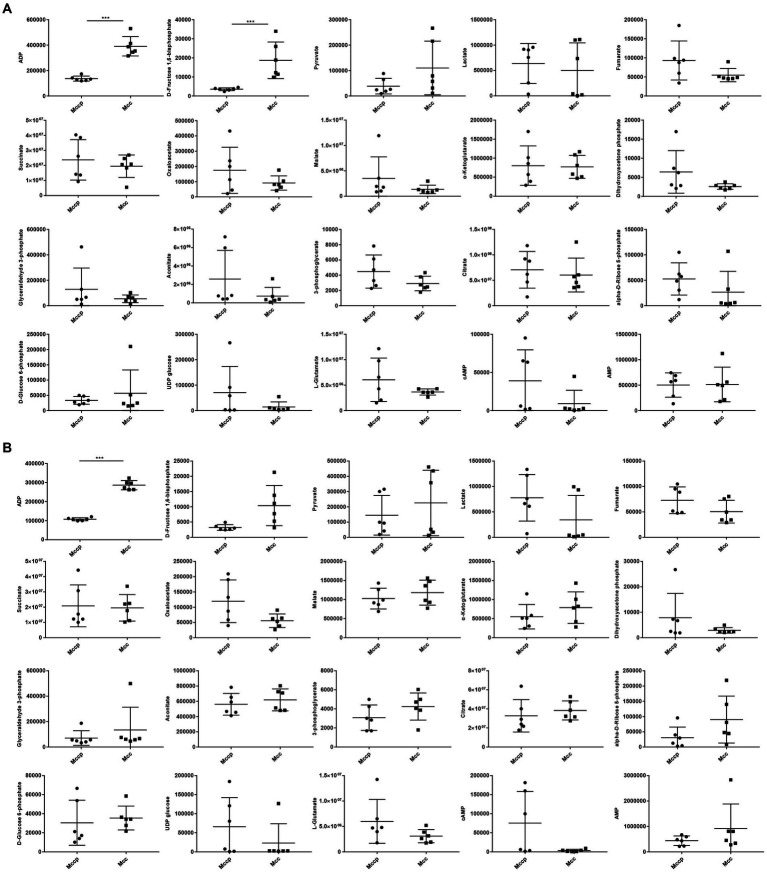
The amount of 20 energy metabolites in Mccp and Mcc in the middle **(A)** and late **(B)** logarithmic phases.

To further verify different metabolites between the Mccp and Mcc groups, targeted metabolomics and MRM technology were used to quantitatively analyze the energy metabolite content of the two *Mycoplasma* species in different periods. The QC samples were prepared by equally mixing all samples and were used to evaluate data stability and repeatability. When the relative standard deviation (RSD) in the QC sample was <30%, the data were considered stable and reliable (Supplementary Figure S2). In total, 20 energy metabolites were detected in the samples (Table 1; Supplementary Table S3), and the content of these metabolites differed between the two *Mycoplasma* species. The content of fructose 1,6-bisphosphate and ADP in the middle logarithmic phase and the content of only ADP in the late logarithmic phase of Mcc significantly increased compared with those in Mccp. The other energy metabolites differed between the two groups. In the middle logarithmic phase, the abundance of three metabolites (glyceraldehyde 3-phosphate, 3-phosphoglycerate, and ribose 5-phosphate) was higher in Mccp than in Mcc. By contrast, in the late logarithmic phase, the abundance was lower in Mccp than in Mcc. The abundance of the middles involved in glycolysis and the TCA cycle exhibited opposite results. In Mccp, the citrate content decreased significantly and the content of several metabolites in the TCA cycle decreased in the late phase compared with the middle phase, whereas the pyruvate content increased dramatically (Supplementary Table S4). Interestingly, lactate accumulation occurred throughout the whole exponential growth phase of Mccp, but not of Mcc. This result is consistent with the previous results of non-targeted metabolomics (Figure 6).

**Table 1:** The list of energy metabolites involved in Mccp and Mcc metabolism.

Component Name	ESI Mode	KEGG ID	Transition	RT(min)	FC(Mccp/Mcc M*)	*p* value	FC(Mccp /Mcc L*)	*p* value
ADP	Negative	C00008	426/134	11.4	0.349	0.000	0.376	0.000
Fructose 1,6-bisphosphate	Negative	C05378	339/241	14.5	0.190	0.003	0.309	0.025
Pyruvate	Negative	C00022	87/43	2.29	0.350	0.141	0.641	0.448
Lactate	Negative	C00256	89/45	5.61	1.272	0.630	2.274	0.140
Fumarate	Negative	C00122	115/71	9.3	1.703	0.112	1.445	0.143
Succinate	Negative	C00042	117/73	9	1.217	0.516	1.065	0.853
Oxaloacetate	Negative	C00036	131/87	10	1.919	0.226	2.148	0.059
Malate	Negative	C00149	133/115	9.8	2.602	0.248	0.869	0.395
a-Ketoglutarate	Negative	C00026	145/101	9.15	1.041	0.899	0.697	0.290
Dihydroxyacetone phosphate	Negative	C00111	169/79.01	11.2	2.503	0.125	2.721	0.238
Glyceraldehyde 3-phosphate	Negative	C00118	169/97.02	1	2.365	0.313	0.523	0.424
Aconitate	Negative	C00417	173/129.01	10.9	3.511	0.192	0.905	0.493
3-phosphoglycerate	Negative	C00197	185/97	11.3	1.540	0.136	0.726	0.176
Citrate	Negative	C00158	191/111	12	1.170	0.620	0.853	0.499
Ribose 5-phosphate	Negative	C00117	229/79	11.2	1.975	0.248	0.340	0.117
Glucose 6-phosphate	Negative	C00092	259/199	11.9	0.587	0.474	0.859	0.658
UDPglucose	Negative	C00029	565/323	11	4.978	0.217	2.864	0.278
L-Glutamate	Positive	C00025	148.1/84.1	10	1.657	0.202	1.942	0.145
cAMP	Positive	C00575	330.06/136	7.2	4.318	0.128	22.847	0.060
AMP	Positive	C00020	348.07/136	10.7	0.977	0.947	0.476	0.257

Blackbody is significantly different, *M means middle of logarithmic growth phase, L means late logarithmic growth phase.

### 3.6 Analysis of metabolites correlation and metabolic pathways

**Figure 7:**
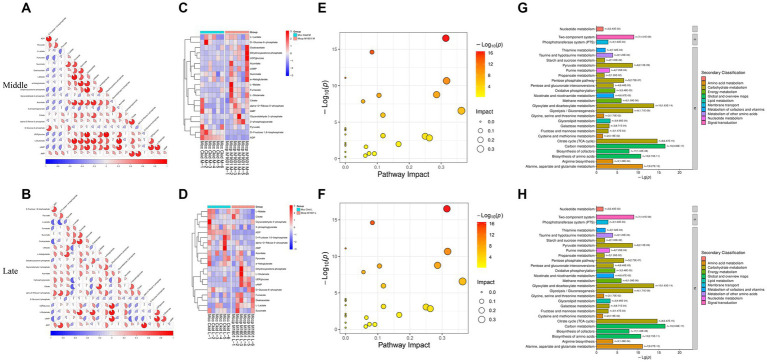
Analysis energy metabolites. The correlation analysis among the significant difference metabolites **(A,B)**. −1 < Pearson’s correlation coefficient (R) < 1. The hierarchical clustering results of significant differential metabolites between Mcc and Mccp **(C,D)**. Bubble plot **(E,F)** and histogram **(G,H)** of KEGG pathways related to the energy metabolites.

**Figure 8:**
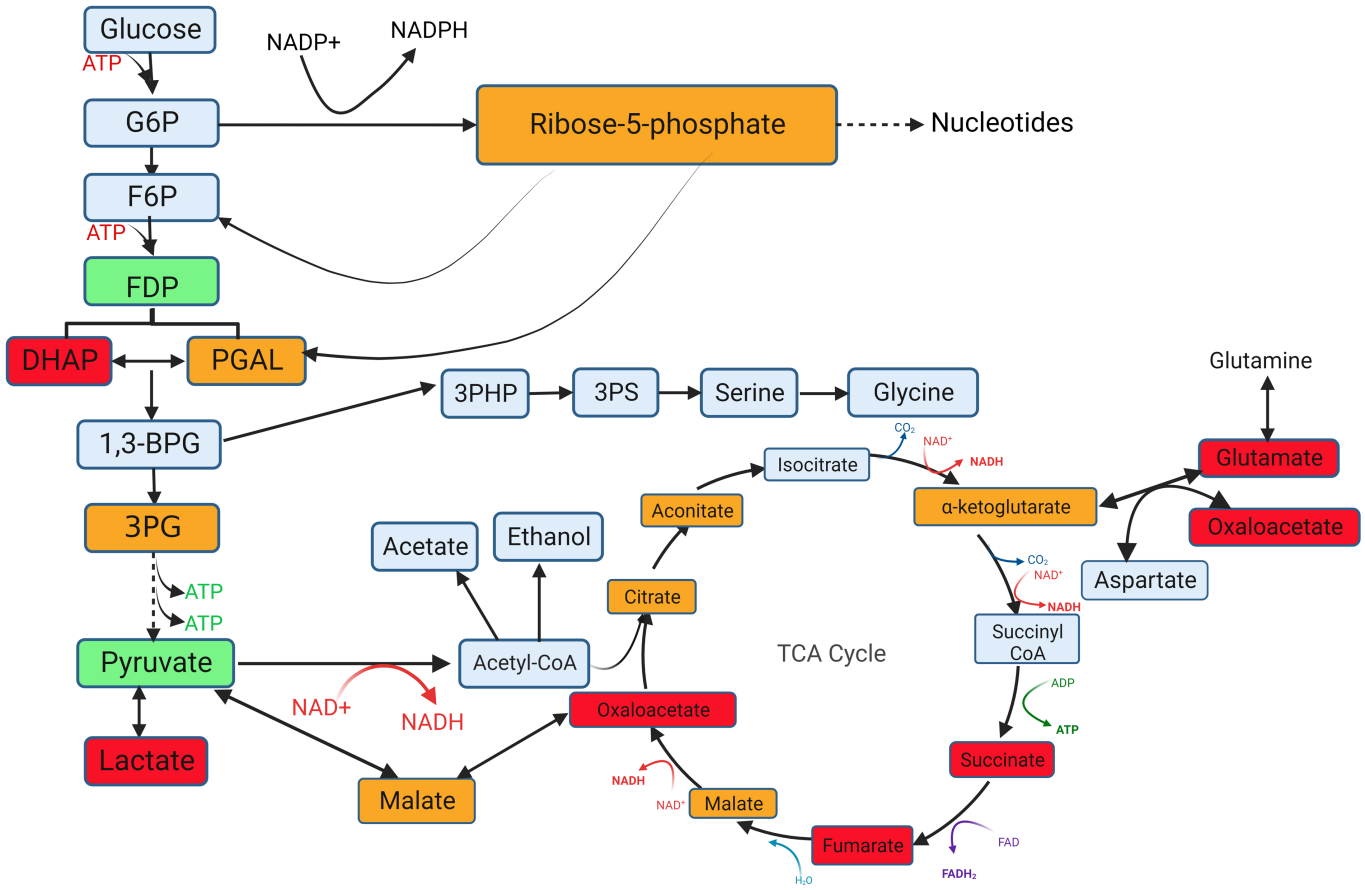
Schematic diagram of differential metabolites in glycolysis, the TCA cycle and PPP obtained by performing KEGG metabolic pathway analysis. G6P, Glucose 6-phosphate; F6P, fructose 6-phosphate; FDP, Fructose-1, 6-diphosphate; DHAP, Dihydroxyacetone phosphate; PAGL, 3-Phosphoglyceraldehyde; 1,3-BPG, 1,3-Disphosphoglycerate; 3-PG, 3-Phosphoglycerate; 3PHP, 3-Phosphonooxypyruvate; 3PS, 3-Phosphoserine. Red indicates that the metabolites were upregulated throughout the logarithmic period, green indicates that they were downregulated during the logarithmic period, yellow indicates that the metabolites were upregulated in the middle logarithmic period and downregulated in the late logarithmic period, and gray represents no change. Created with BioRender.com.

The correlation between different metabolites was analyzed using the PCA method (Figure 7). In the middle stage of logarithmic growth of the Mcc strain, lactate accumulation was the most negatively correlated with pyruvate and the most positively correlated with succinate. However, in the late stage of the rapid growth period, lactate concentration was the most negatively correlated with fructose 1,6-bisphosphate and the most positively correlated with oxaloacetate. Pyruvate and fructose 1,6-bisphosphate are crucial metabolites produced during glycolysis/gluconeogenesis, and succinate and oxaloacetate are middle metabolites of the TCA cycle. This indicates that lactate was related to the efficiency of glycolysis/gluconeogenesis and the TCA cycle in Mcc. Given the high correlation between these metabolites, the biochemical process, that is, the entry of lactate into gluconeogenesis or the TCA cycle through conversion to pyruvate, was speculated to be more active in Mcc than in Mccp or does not exist in Mccp, or less lactate was produced. At the same time, pyruvate exhibited the strongest positive correlation with ADP in the middle stage and with glucose 6-phosphate in the late stage. This also indicated that gluconeogenesis plays a role during the logarithm phase. To further explore the metabolic pathways involved in different metabolites in each group, energy metabolites were submitted to the KEGG online website^5^ for the analysis and enrichment of related metabolic pathways. The differential metabolites were mainly enriched in glycolysis, TCA cycle, pentose phosphate pathway (PPP), and nucleotide metabolism (Figure 8). Pyruvate, generated from glucose through the glycolytic pathway, is a key middle in the network of metabolic pathways. The pyruvate content might increase due to increased glycolysis or reduced catabolic capacity of the TCA cycle. Pyruvate was higher in Mcc than in Mccp during the entire logarithmic period, indicating that the glycolytic pathway in Mcc was more active.

### 3.7 HEPES buffer system improves Mccp growth

**Figure 9:**
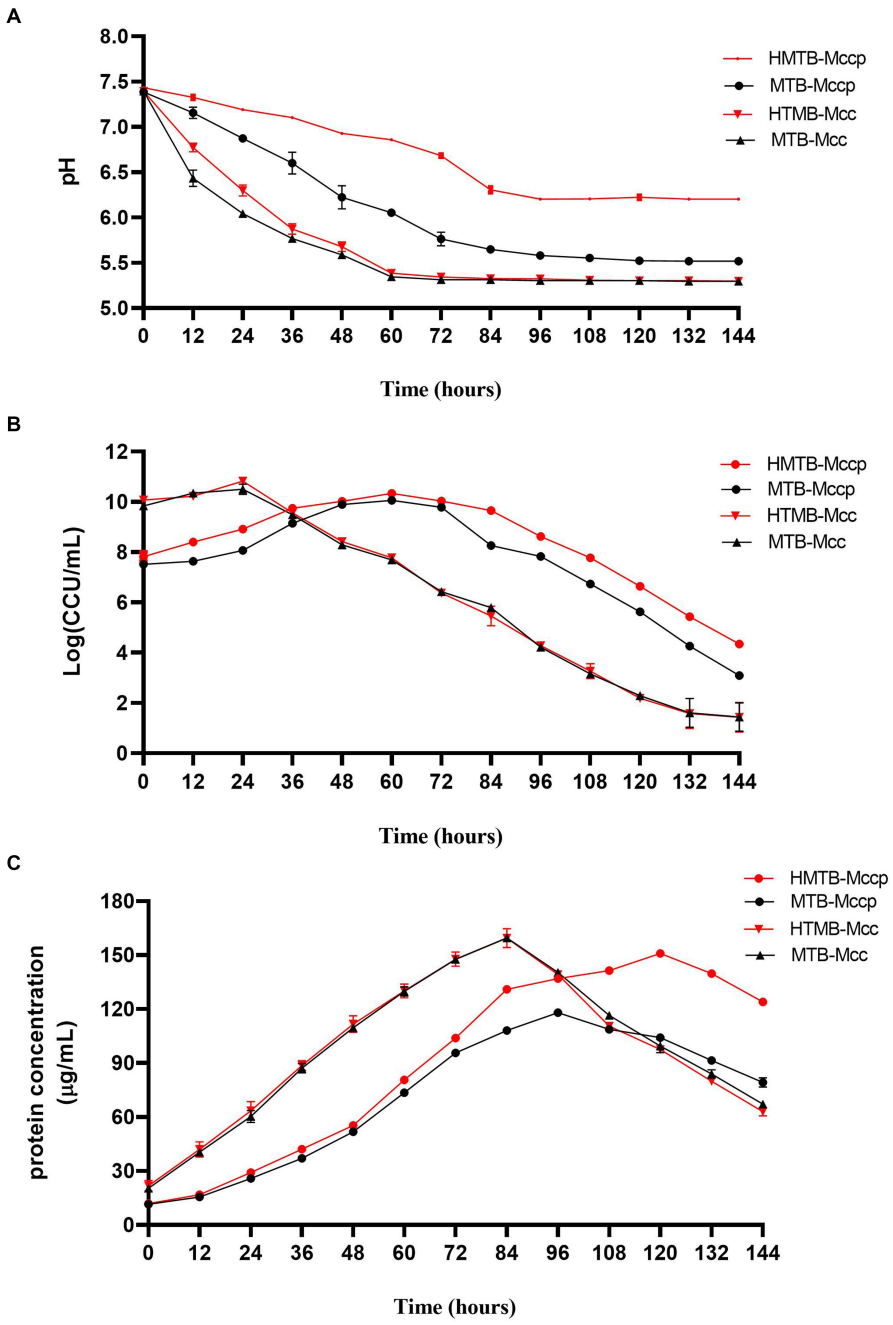
HEPES-buffered broth improves Mccp growth. The media pH **(A)**, bacterial titer **(B)**, and protein concentration **(C)** during the growth of Mcc and Mccp in MTB and HEPES-buffered broth (HMTB).

Based on the metabolomics analysis of lactate accumulation that occurred during the entire logarithmic phase of Mccp growth, and the culture pH that reduced faster and considerably more than that of Mcc, N-[2-hydroxyethyl]piperazine-N′-[2-ethanesulfonic acid] (HEPES) was added to the culture medium to evaluate its effect on Mccp and Mcc growth. With 20 mM HEPES addition, the rapid decrease in the culture pH for Mccp was effectively prevented and no obvious effect was observed on the culture pH for Mcc. For Mccp, the maximum protein content was 117 μg/mL at 96 h after inoculation, and this declined to 79 μg/mL at 144 h. Only 10^3^ CCU/mL viable bacteria (Mccp) could be detected in conventional MTB. The protein content was always greater than 120 μg/mL between 84 and 144 h, and the maximum content was 151 μg/mL at 120 h in HEPES-buffered broth (HMTB). The bacterial titer of Mccp in HMTB was higher than that of MTB in the growth stage (0~114 h) (Figure 9). The stationary period was extended and the decrease in the count of bacteria cell slowed down, thereby prolonging the time and amount of protein harvest. The results revealed that HEPES improved the bacterial titer and protein content for Mccp, and the improvement was not obvious for Mcc.

## 4 Discussion

Metabolism is essential for microbial survival, including catabolism to generate biologically usable energy and consume energy to synthesize cellular components for growth. Glycolysis is a central metabolic pathway of organisms, which not only decomposes nutrients to contribute energy for growth but also provides precursors for the synthesis of other compounds (Wolfe, 2015). Meanwhile, the TCA cycle and PPP are also indispensable energy metabolism pathways. In this study, based on the diversity in the growth phenotype between the two *Mycoplasma* species Mccp and Mcc, the differential metabolites between them were identified through non-targeted metabolomics and targeted energy metabolites. Both middle and late stages of rapid growth were selected for more detailed comparison.

The concentration of intermediary metabolite in the TCA cycle and PPP was downregulated in the middle stage and upregulated in the late stage for Mcc. This suggested that the TCA cycle and PPP in Mcc became active after the middle logarithmic phase. Before this phase, glucose was mainly used for generating ATP through glycolysis for cell growth, and then, it was oxidized through PPP to generate 5-phosphoribose and NADPH for nucleic acid synthesis and reductive biosynthesis, respectively. For Mccp, before the logarithmic phase, the metabolic flow direction of glucose is more inclined in the PPP direction, that is, 6-phosphate generated through glucose phosphorylation was oxidized and rearranged to produce ribose-5-phosphate. This compound may be stored as a five carbon sugar raw material for nucleic acid synthesis and can be used for later growth. The TCA cycle in Mccp may be active before the middle logarithmic period because it absorbed pyruvate from the medium that was entering the TCA pathway. However, the metabolic processes of the TCA cycle and pentose phosphate pathway in Mcc may require pyruvate accumulation to a certain extent. This is consistent with observations that media enriched with 0.2% (up to 0.8%) pyruvate perform considerably better in primary isolation and antigen production for Mccp (WOAH, 2021) while not for Mcc. Isotope labeling studies have shown that *M. bovis* primarily produces phosphorylated sugars through gluconeogenesis, rather than through absorption of exogenous glucose, even when the exogenous glucose level was high. The absorption and glycolysis of exogenous glucose in *M. bovis* were lower than that in *M. gallisepticum*. By contrast, *M. gallisepticum* actively absorbs exogenous glucose and accumulates a large intracellular pool of hexose phosphate, which is subsequently catabolized during glycolysis and PPP (Masukagami et al., 2017). The metabolic flow direction of Mccp may be similar to that of *M. bovis* and needs to be verified further in the isotope tracer test. It is widely accepted that false positive identifications are a significant challenge for untargeted metabolomic (Schrimpe-Rutledge et al., 2016), the metabolites identified in the untargeted metabolomics need further be validated by targeted metabonomics analysis. It is worth noting that old Mcc strain Ckid was used to compare with Mccp M1601 in the present study. The metabomomics may be different for old and new strains and need to investigate to better understanding their metabolomic characteristics.

Mycoplasmas lack a cell wall, and so are more sensitive to environmental changes than other bacteria. *Mycoplasma* species vary in their sensitivity levels. The growth medium pH is a key factor affecting bacterial growth and viability. The HEPES buffer system in conjunction with the standard Gourlay’s culture medium could effectively prevent the decline in the pH of *M. mycoides* subsp. *mycoides* culture medium, increase the titer of viable bacteria, and considerably prolong culture survival (Waite and March, 2001). In a study on *M. pneumoniae*, at physiological (high) pH, generated ATP was considerably used for growth compared with at low pH, while reversible metabolic stalling was induced by an unfavorable ion balance for ATP generation in an acidic medium (Wodke et al., 2013). Lactate accumulation occurred in the entire logarithmic period of Mccp, resulting in low pH, which was not favorable for rapid growth. This was consistent with the result that the pH of the Mccp growth medium rapidly descended to approximately 5.4, even before the middle logarithmic period, while the pH of the Mcc growth medium was still above 6.5 at the end of the logarithmic phase. The stationary phase of Mccp was prolonged, and the protein concentration was increased by 30–40% when Mccp was grown in the buffer medium supplemented with HEPES. With HEPES addition, the pH of the growth medium decreased slowly compared to that of the unbuffered medium. This indicated that HEPES addition could offer a relatively stable pH environment for bacterial growth, thus alleviating the pressure of the lactate accumulation-induced acidic environment and improving the Mccp titer. Thus, HEPES addition to growth medium is useful for Mccp antigen production in vaccine development.

## 5 Conclusion

*Mycoplasma* species Mcc and Mccp are both significant economical crucial pathogens. They share a high degree of genome homology with each other. In this study, differential metabolite profiles were identified in these two species through untargeted and targeted metabonomics analysis. Compared with Mccp, the content of fructose 1,6-bisphosphate, ADP, and pyruvate was abundant in Mcc during the whole logarithmic period. Lactate abundance in Mccp may be related to low pH, slow growth, and low bacterial viability and may be improved with HEPES addition. The metabolic characteristics analysis of the two significant *Mycoplasma* species will be helpful in understanding their pathogenesis and developing mycoplasma culture medium.

## Data availability statement

The original contributions presented in the study are included in the article/Supplementary material. The data presented in the study are deposited in the MetaboLights repository, accession number MTBLS8542.

## Ethics statement

The animal study was approved by the Animal Ethics Committee of Lanzhou Veterinary Research Institute, Chinese Academy of Agricultural Sciences. The study was conducted in accordance with the local legislation and institutional requirements.

## Author contributions

HH: Conceptualization, Data curation, Formal analysis, Funding acquisition, Investigation, Methodology, Project administration, Resources, Software, Validation, Visualization, Writing – original draft, Writing – review & editing. XZ: Data curation, Formal analysis, Investigation, Methodology, Visualization, Writing – original draft. SC: Data curation, Formal analysis, Investigation, Visualization, Writing – original draft, Writing – review & editing. SLan: Data curation, Formal analysis, Writing – review & editing. ZL: Data curation, Formal analysis, Writing – review & editing. SLiu: Data curation, Formal analysis, Writing – review & editing. XY: Data curation, Formal analysis, Writing – review & editing. PG: Data curation, Formal analysis, Writing – review & editing. YC: Conceptualization, Data curation, Project administration, Supervision, Writing – review & editing.

## Footnotes

^1^ https://xcmsonline.scripps.edu/index.php

^2^ https://www.metaboanalyst.ca

^3^ https://www.kegg.jp

^4^ https://hmdb.ca/

^5^ http://www.omicsbean.cn/
