# Supplementary material for: Comparative untargeted and targeted metabonomics reveal discriminations in metabolite profiles between Mycoplasma capricolum subsp. capripneumoniae and Mycoplasma capricolum subsp. capricolum
Source: Front Microbiol. 2023 Dec 8;14:1294055. doi: 10.3389/fmicb.2023.1294055 (PMC10740972; doi:10.3389/fmicb.2023.1294055)
Supplement: Supplementary file 5 [file Data_Sheet_1.DOCX]

***Supplementary Material***

**1 Supplementary Figures and Tables**

**1.1 Supplementary Figures**

**Figure S1**. The TIC overlap pattern of the positive (A) and negative (B) ion modes of the quality control samples.

**Figure S2.** The relative standard deviation distribution of energy metabolites of the quality control samples in quantitative analysis.

**1.2 Supplementary Tables**

**Table S1**. The ion peaks of all metabolites.

**Table S2.** The chromatographic peak area of Mccp and Mcc on energy metabolites.

**Table S3.** Comparison of energy metabolites in the intermediate and late stages of the logarithmic period.
