# Supplementary figures and images for: Comparative untargeted and targeted metabonomics reveal discriminations in metabolite profiles between Mycoplasma capricolum subsp. capripneumoniae and Mycoplasma capricolum subsp. capricolum
Source: Front Microbiol. 2023 Dec 8;14:1294055. doi: 10.3389/fmicb.2023.1294055 (PMC10740972; doi:10.3389/fmicb.2023.1294055)

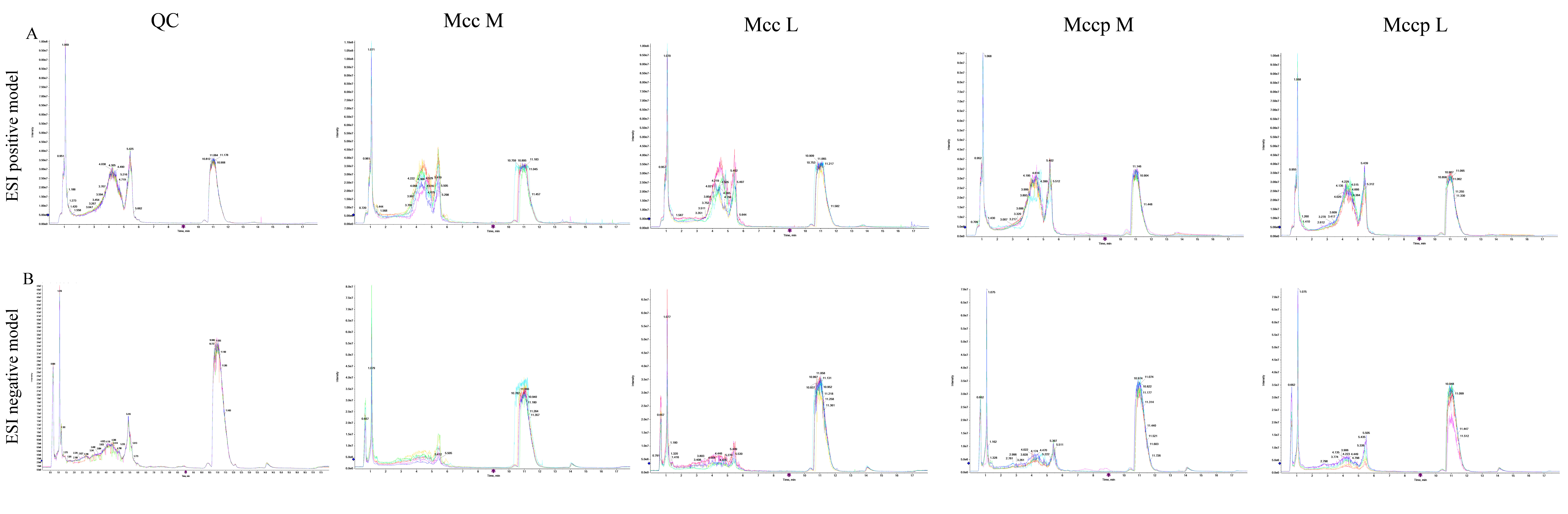

Supplement: Supplementary file 6 [file Image_1.JPEG]

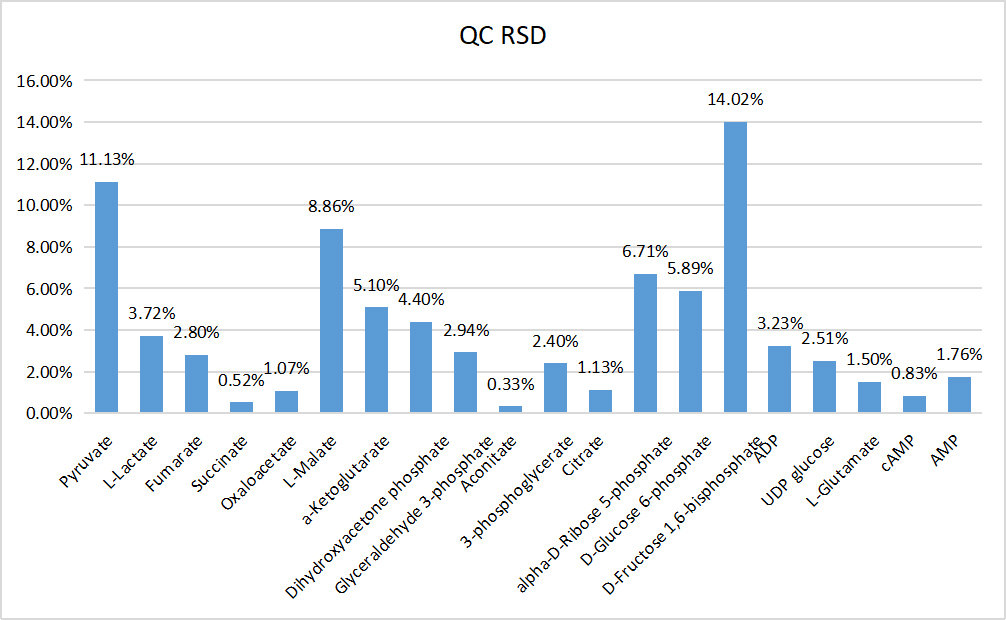

Supplement: Supplementary file 7 [file Image_2.JPEG]
